# Health-Related Outcomes of Adverse Childhood Experiences in Texas, 2002

**Published:** 2010-04-15

**Authors:** Shanta R. Dube, Michelle L. Cook, Valerie J Edwards

**Affiliations:** Centers for Disease Control and Prevention, National Center for Chronic Disease Prevention and Health Promotion; Center for Health Statistics, Texas Department of State Health Services, Austin, Texas; National Center for Chronic Disease Prevention and Health Promotion, Centers for Disease Control and Prevention, Atlanta, Georgia

## Abstract

**Introduction:**

We assessed the prevalence of 7 childhood adversities (psychological, physical, and sexual abuse; household mental illness; household substance abuse; maternal battery; and incarceration of a household member) and the associations of those adversities with health outcomes.

**Methods:**

Using data from 5,378 people who responded to the 2002 Texas Behavioral Risk Factor Surveillance System survey (which included questions about childhood adversity), we created 4 groups: no childhood abuse or household dysfunction, childhood abuse only, household dysfunction only, and both childhood abuse and household dysfunction. We examined groups by sociodemographic variables and the association with current smoking, obesity, and self-rated health.

**Results:**

Among adult respondents, 46% reported at least 1 childhood adversity. Reports of both household dysfunction and abuse were significantly lower for college graduates than for people with less education. For those with both abuse and household dysfunction, the odds of current smoking were 1.9 and for obesity were 1.3. Compared to people without childhood adversities, people who experienced childhood adversities more frequently reported having fair or poor general health status.

**Conclusion:**

Childhood adversities are common among Texas adults. People with childhood adversities are more likely to be socioeconomically disadvantaged, less educated, and have difficulties maintaining employment in adulthood compared to people with no adversities. Moreover, childhood adversities appear to be associated with health problems such as current smoking, obesity, and poor or fair general health among Texas adults.

## Introduction

In 2002, child protective service agencies confirmed that more than 900,000 children in the United States had suffered maltreatment (1). Among those children, 61% had experienced neglect; 19%, physical abuse; 10%, sexual abuse; and 5%, emotional or psychological abuse. Furthermore, an estimated 1,500 children were confirmed to have died from maltreatment; 36% of these deaths were estimated to be from neglect, 28% from physical abuse, and 29% from multiple types of maltreatment (1). These data are derived from cases reported to authorities; however, a large proportion of childhood abuse and childhood exposure to serious dysfunction goes undetected and unreported (2-4). Thus, retrospective reports from adult survivors of childhood abuse and serious family dysfunction may help illuminate the effect and lifetime health sequelae of these experiences.

Scientific evidence is mounting that such adverse childhood experiences (ACEs) have a profound long-term effect on health. Research shows that exposure to abuse and to serious forms of family dysfunction in the childhood family environment are likely to activate the stress response, thus potentially disrupting the developing nervous, immune, and metabolic systems of children (5-7). ACEs are associated with lifelong physical and mental health problems that emerge in adolescence and persist into adulthood, including cardiovascular disease, chronic obstructive pulmonary disease, autoimmune diseases, substance abuse, and depression (8-12).

We used data from the 2002 Texas Behavioral Risk Factor Surveillance System (BRFSS) to calculate and report prevalence estimates for 7 ACEs and for specific groupings: no abuse or household dysfunction, household dysfunction only, abuse only, and both household dysfunction and abuse. The proportion of people reporting ACEs was also examined by select sociodemographic variables. Furthermore, we examined the associations between ACEs and smoking, body mass index (BMI), and self-rated health.

## Methods

The Texas BRFSS is an ongoing, state-based telephone survey by landline that collects information from noninstitutionalized adults aged 18 years or older on health risk behaviors, preventive health practices, and access to and use of health care services primarily related to chronic conditions. In 2002, Texas administered a set of state-added questions on the BRFSS questionnaire to assess ACEs among adults. The questions about adverse experiences that occurred before age 18 covered emotional, physical, and sexual abuse; growing up with domestic violence, substance abuse, and mental illness; and any history of an incarcerated family member. In 2002, the Council of American Survey Research Organizations (CASRO) response rate was 46%. Among the 6,107 respondents who participated in the Texas BRFSS survey, the responses of 542 were excluded because of missing data.

### ACEs

In 2002, the Texas Department of State Health Services added 17 questions (Table 1) derived from the Centers for Disease Control and Prevention (CDC)–Kaiser Permanente ACE Study (13) to its BRFSS survey. Because of the sensitivity of the questions, the ACE questions were included at the end of the survey. Question-by-question refusals were minimal, ranging from 0.5% to 1.0%. Of the 6,107 respondents, 5,378 (88%) answered all 17 ACE questions. All questions about ACEs pertained to the respondents' first 18 years of life (≤18 years of age). Questions on psychological and physical abuse were adapted from an earlier scale (14). Contact sexual abuse items were adapted from a scale developed by Wyatt (15). In addition, 4 exposures to household dysfunction during childhood were assessed: household exposure to substance abuse, mental illness, mother or stepmother treated violently (14), and someone in the household having been incarcerated. Each question had a yes or no response.

We created 7 categories of ACEs based on the 17 questions: psychological abuse, physical abuse, sexual abuse, substance abuse, mental illness, mother or stepmother treated violently, and criminal behavior in the household. Because ACEs tend to co-occur (16-18), we created 2 separate categories of exposures by combining the 7 individual ACEs: childhood abuse and household dysfunction. For analysis purposes, we grouped the 5,378 respondents as follows: no childhood abuse or household dysfunction (n = 2,916), childhood abuse only (484), household dysfunction only (1,001), and both childhood abuse and household dysfunction (977).

### Health behavior outcomes

We examined 3 health outcomes in relation to ACEs: current smoking, obesity, and self-reported general health. Current smoking was defined as having ever smoked 100 cigarettes and currently smoking some days or every day. Obesity was defined as having a BMI of ≥30 kg/m^2^. Self-reported general health was assessed by asking, "Would you say that in general your health is excellent, very good, good, fair, or poor?" We created a 2-level variable by collapsing "fair" or "poor" into 1 group and "excellent," "very good," or "good" into a second group. People reporting good to excellent health served as the reference group in multivariate logistic regressions.

### Data analysis

Data were analyzed in SPSS version 13.0 (SPSS, Inc, Chicago, Illinois) and SUDAAN version 9.0.1 (Research Triangle Institute, Research Triangle Park, North Carolina). The proportion of people reporting ACEs was examined by selected sociodemographic variables: age, race/ethnicity, sex, education level, annual household income, and employment status. We weighted the estimates to adjust for the probabilities of selection; we used a poststratification weighting factor to represent the distribution of Texas adults by age and sex at the state level. Since there were clear differences between ACEs by age group, all reported prevalence estimates were age-adjusted to the 2000 US census population using 6 age categories (18-24, 25-34, 35-44, 45-54, 55-64, and 65 years or older). We obtained odds ratios using multivariate logistic regressions; covariates in all models included age, sex, race/ethnicity, education level, and employment status. An α of .05 was used for the test of significance in the logistic regression analyses.

## Results

Among adult Texans, slightly more than one-fourth (27%) reported any form of childhood abuse, and 37% reported exposure to any form of household dysfunction (Table 1). Nearly half (46%) of all adult Texans had at least 1 ACE.

### ACE groupings by demographic characteristics

The prevalence of ACEs did not vary by race/ethnicity or sex for any of the 4 categories (Table 2). Differences were noted by education level; reports of those experiencing both household dysfunction and childhood abuse were lower for college graduates (13%) than for people with some college, high school graduates, or those who did not graduate from high school.

Differences were also noted for annual household income and employment status. The proportion reporting both household dysfunction and childhood abuse was lowest among those who had household income of at least $50,000, compared to adult Texans with income of $25,000 to $49,999 or income less than $25,000. The proportion reporting both household dysfunction and childhood abuse among people unable to work was higher than for people employed for wages and homemakers.

### ACEs and smoking, obesity, and general health

Almost one-third (32%) of adults in Texas who experienced both childhood abuse and household dysfunction reported current smoking (Figure). The prevalence of obesity was higher among those who experienced both household dysfunction and childhood abuse and those who experienced childhood abuse only compared with those who did not report any ACEs. Fair or poor general health status was more frequently reported among people who grew up in a dysfunctional household, were abused as a child, or had at least 1 experience in both categories of ACEs.

**Figure:**
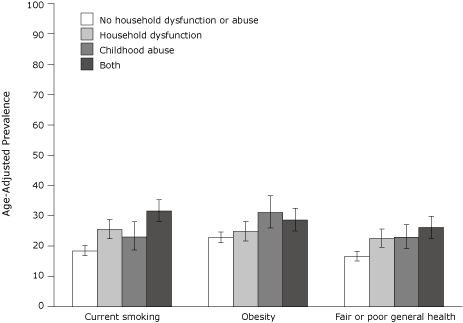
Age-adjusted prevalence rates of current smoking, obesity, and fair or poor general health by adverse childhood experience categories, Texas, 2002. The bars represent 95% confidence intervals. Data are from the 2002 Texas Behavioral Risk Surveillance System, age-standardized to the 2000 US census. Current smoking was defined as smoking at least 100 cigarettes and now smoking some days or every day. Obesity was defined as having a body mass index of ≥30 kg/m^2^. Fair or poor self-reported general health was defined as reporting "fair" or "poor" compared with "excellent," "very good," or "good." Childhood abuse was determined through responses to questions regarding psychological, physical, and sexual abuse. Household dysfunction was determined through responses to questions regarding substance abuse, mental illness, whether mother or stepmother was treated violently, and incarceration of a household member.

**Table:** 

**Outcome**	**No Household Dysfunction or Childhood Abuse, % (95% CI)**	**Household Dysfunction Only, % (95% CI)**	**Childhood Abuse Only, % (95% CI)**	**Both Household Dysfunction and Childhood Abuse, % (95% CI)**
**Current smoker**	18.4 (16.8-20.2)	25.5 (22.4-28.8)	23.0 (18.7-28.0)	31.6 (28.1-35.3)
**Obesity**	22.8 (21.1-24.6)	24.8 (21.7-28.1)	31.1 (26.0-36.6)	28.6 (25.0-32.5)
**Fair or poor general health**	16.6 (15.1-18.3)	22.5 (19.6-25.6)	22.9 (19.2-27.1)	26.1 (22.6-29.8)

Compared to people with no ACEs, there was a 40% increased likelihood of current smoking for people who reported any household dysfunction and a 90% increased likelihood for people with both household dysfunction and childhood abuse (Table 3). Compared to people with no ACEs, the odds for obesity increased by 50% among people who reported any childhood abuse and increased by 30% among people reporting both childhood abuse and household dysfunction. People who grew up with any childhood abuse, any household dysfunction, or both were more likely to report fair or poor general health than were people with no ACEs.

## Discussion

The questions in the 2002 Texas BRFSS provided a unique opportunity to obtain and report estimates for 7 categories of ACEs in a population-based sample. Our findings demonstrate that abuse and household dysfunction are common, and the findings are similar to findings from the CDC–Kaiser Permanente ACE Study (12) among adult health maintenance organization members, in which 56% reported experiencing at least 1 ACE.

Using data obtained from the Texas BRFSS, we were also able to examine the prevalence of ACEs by sociodemographic characteristics. We observed a higher prevalence of ACEs among people with lower levels of education and a gradient in the proportion of ACEs by education level. Similarly, we observed a higher burden of ACEs for people with lower annual household income compared with those in the higher income brackets and for people unable to work compared with people employed for wages. These findings demonstrate that childhood adversity may be disproportionately represented in segments of the population where health disparities are often observed, and they suggest that adverse experiences in childhood may be associated with lower socioeconomic status later in life.

Among adults who experienced abuse or were exposed to serious dysfunction, a higher percentage reported being current smokers compared with people reporting no ACEs, similar to findings in a previous study (19). In another study in 4 separate birth cohorts dating back to 1900 (20), childhood stressors were associated with a lifetime history of smoking, which demonstrates that the childhood family milieu may be a salient factor to consider, despite any influences of social and secular trends to change the behavior during the past century.

Among adults who reported any form of childhood abuse, there was a higher proportion of people with a BMI ≥30 kg/m^2^ compared with people who did not report a history of abuse or household dysfunction in childhood, even after adjusting for sociodemographic factors. In a weight loss program conducted by Kaiser Permanente, clinicians and researchers discovered that sexual abuse in childhood was common among adult program dropouts and that abuse predated obesity (13). Severity of abuse was also associated with obesity in the Kaiser cohort (21). Our observations help us understand the potential contributing role of ACEs to overweight and obesity in adulthood.

Self-reported poor or fair general health among Texas adults was more prevalent among people reporting household dysfunction, childhood abuse, or both compared with people reporting no ACEs, and the association persisted even after controlling for sociodemographic factors. This is not surprising, given that people who report ACEs may have a wide variety of physical and mental health problems. Prior studies support this possibility: people who experienced childhood adversity had lower scores across all 8 domains of health-related quality of life, as measured by the Standard Form-36 (22). There is evidence that exposure to ACEs is related to difficulties in emotional self-regulation, which may lead to reports of fair or poor general health (23,24).

Epidemiologic studies documenting the associations between childhood adversity and negative health outcomes in adulthood are converging with studies in the neurosciences that have documented physiological and anatomical changes in the brains of people who experienced childhood abuse. These studies may provide biological plausibility for our findings (25). For example, a study that used electroencephalograms to measure limbic irritability (7) found a higher percentage of clinically significant brain-wave abnormalities among people who had a history of early trauma than among those who did not experience early trauma (7). Magnetic resonance imaging has revealed reductions in hippocampal volumes among severely sexually abused women, and reductions in the intracranial and cerebral volumes among maltreated children compared with those who were not maltreated (5,6). Additionally, the limbic system, which is responsible for emotional response, is adversely affected (5).

Anatomic and functional neurologic changes may occur among people who experienced 1 or more forms of abuse compared with nonabused people (5,6,26) through repeated or chronic activation of the stress response. The relationships we observed between specific ACE groupings with smoking, obesity, and self-rated health may indicate the inherent human stress response (27); effects of the adrenal release of catecholamines and corticosteroids on developing neurons and neural networks is a biologic phenomenon that cannot be ruled out as a mechanism for the associations observed.

Several limitations should be considered when interpreting these results. First, the BRFSS is a telephone survey by landline and not all people in the United States have landline telephones. This could limit the generalizability of the results of this survey. Although the Texas BRFSS data are representative of the Texas population, they differ from that of the US population and, therefore, generalizability to the US population is limited. For example, Texas has a higher percentage of foreign-born people, people who speak a language other than English at home, and families living below poverty level compared with the US population. Texas also has a lower percentage of adults who graduate high school, people aged 18 years or older, adults aged 65 years or older, and people who self-identify as Hispanic or Latino compared with the overall US population. Second, the responses to BRFSS questions are self-reports, and independent verification of reported exposures is not possible. However, longitudinal follow-up of adults whose childhood abuse was documented has shown that their retrospective reports of their experiences are likely to underestimate actual occurrence (28,29). Moreover, the test-retest reliability of retrospective reports of ACEs from adults was in the good-to-excellent range (30). In addition, because self-reported general health is subjective, it is possible that it is providing a measure of psychological well-being.

The method of questionnaire administration may have led to underreporting of exposures. Disclosure of sensitive topics (such as childhood abuse) to a stranger conducting a telephone survey may result in a downward bias in estimating prevalence compared with surveys in which such disclosure was obtained in a more private manner. We did find some differences between the prevalence estimates from the CDC–Kaiser Permanente ACE Study (13) and the BRFSS results. For instance, the prevalence of growing up with substance abuse in the home was somewhat lower than in other national surveys. Studies have reported that 1 in 4 of the adult population report growing up in homes with alcoholism (31); our study found that 1 in 5 reported this exposure. Similarly, the prevalence of sexual abuse (8%) in our analysis is substantially lower than most prevalence estimates, which have ranged from 15% to 25% (32,33). Finally, some of the estimates had large confidence intervals due to small sample sizes, indicating that caution must be used when interpreting the findings.

Despite these limitations, the findings suggest that growing up with abuse and serious forms of family dysfunction among adults in Texas is common. The findings also highlight the health effects associated with ACEs among adults in Texas. Continued public health attention is needed to prevent child abuse and concomitant stressful family exposures and to address ACEs in association with health problems. Such efforts will lead to improved well-being in the nation as a whole.

## Figures and Tables

**Table 1:** Age-Adjusted Prevalence of Adverse Childhood Experiences, 2002 Texas Behavioral Risk Factor Surveillance System^a^

Adverse Childhood Experiences	Prevalence (95% Confidence Interval)
**Psychological abuse** Did a parent or other adult in the household. . . 1. Often or very often swear at you, insult you, or put you down? 2. Often or very often act in a way that made you afraid that you would be physically hurt?	19.4 (18.2-20.7)
**Physical abuse** Did a parent or other adult in the household. . . 1. Often or very often push, grab, slap, or throw something at you? 2. Often or very often hit you so hard that you had marks or were injured?	13.7 (12.7-14.9)
**Sexual abuse** Did an adult or person at least 5 years older than you ever. . . 1. Touch or fondle you in a sexual way? 2. Have you touch their body in a sexual way? 3. Attempt oral, anal, or vaginal intercourse with you? 4. Actually have oral, anal, or vaginal intercourse with you?	8.7 (7.9-9.5)
**Any childhood abuse**	26.7 (25.4-28.1)
**Household substance abuse** During your childhood did you. . . 1. Live with anyone who was a problem drinker or alcoholic? 2. Live with anyone who used street drugs?	20.7 (19.4-22.0)
**Mental illness in household** 1. Was a household member depressed or mentally ill? 2. Did a household member attempt suicide?	19.3 (18.1-20.5)
**Mother treated violently** Was your mother or stepmother. . . 1. Sometimes, often, or very often pushed, grabbed, slapped, or had something thrown at her? 2. Sometimes, often, or very often kicked, bitten, hit with a fist, or hit with something hard? 3. Ever repeatedly hit over at least a few minutes? 4. Ever threatened with or hurt by a knife or gun?	10.5 (9.6-11.5)
**Incarcerated household member** 1. Did a household member go to prison?	8.2 (7.3-9.1)
**Any household dysfunction**	36.9 (35.5-38.5)

a Prevalence estimates are age-standardized to the US 2000 census data..

**Table 2:** Age-Adjusted Prevalence of Adverse Childhood Experiences, by Demographic Characteristics, 2002 Texas Behavioral Risk Factor Surveillance System^a^

**Characteristic**	n^b^	No Household Dysfunction or Childhood Abuse, % (95% CI)	Household Dysfunction^c^ Only, % (95% CI)	Childhood Abuse^d^ Only, % (95% CI)	Both Household Dysfunction and Childhood Abuse, % (95% CI)
**Total**	5,352	54.3 (52.7-55.8)	19.3 (18.1-20.6)	8.8 (8.0-9.7)	17.7 (16.5-18.9)
**Race/ethnicity**					
White	3,425	53.0 (51.0-54.9)	19.3 (18.0-21.1)	9.3 (8.3-10.5)	18.0 (16.5-19.5)
Black	456	55.1 (49.5-60.5)	19.6 (15.5-24.3)	8.8 (6.3-12.3)	17.1 (13.3-21.6)
Hispanic	1,242	55.0 (51.5-58.6)	20.1 (17.3-23.2)	7.5 (5.9-9.6)	18.6 (16.0-21.5)
Other	197	61.6 (53.4-69.2)	14.1 (9.7-20.8)	8.4 (4.8-14.3)	15.3 (9.8-23.0)
**Sex**					
Men	2,102	55.2 (52.8-57.6)	19.3 (17.4-21.2)	9.1 (7.8-10.6)	16.4 (14.6-18.4)
Women	3,250	53.3 (51.4-55.3)	19.3 (17.7-21.0)	8.6 (7.6-9.8)	18.8 (17.3-20.4)
**Education level**					
Less than high school	833	51.3 (47.2-55.4)	22.4 (19.0-26.1)	7.7 (6.0-9.9)	18.6 (15.7-21.9)
High school graduate	1,434	52.3 (49.4-55.3)	19.1 (16.8-21.7)	9.7 (8.0-11.8)	18.8 (16.7-21.2)
Some college	1,410	51.6 (48.6-54.6)	19.1 (16.9-21.5)	8.6 (7.0-10.4)	20.7 (18.2-23.6)
College graduate	1,666	60.4 (57.4-63.3)	17.5 (15.3-19.8)	9.0 (7.4-11.0)	13.1 (11.3-15.2)
**Annual household income**					
<$25,000	1,549	51.0 (48.0-54.0)	19.8 (17.5-22.4)	8.4 (6.9-10.3)	20.8 (18.2-23.6)
$25,000-$49,999	1,545	50.5 (47.7-53.4)	20.5 (18.2-23.1)	9.2 (7.6-11.1)	19.7 (17.6-22.1)
≥$50,000	1,672	57.5 (54.4-60.5)	18.9 (16.6-21.4)	9.3 (7.7-11.1)	14.3 (12.3-16.6)
**Employment**					
Employed	2,754	55.7 (53.0-58.4)	19.9 (17.7-22.4)	8.7 (7.4-10.2)	15.6 (14.1-17.3)
Self-employed	498	50.9 (45.0-56.9)	16.6 (13.4-20.5)	10.4 (7.7-13.8)	22.0 (17.3-27.6)
Out of work	305	48.1 (40.4-55.8)	22.0 (16.0-29.5)	9.7 (5.7-16.1)	20.2 (15.4-26.0)
Homemaker	558	61.0 (56.4-65.5)	18.5 (15.0-22.6)	7.2 (5.1-10.0)	13.3 (10.6-16.6)
Student	199	41.1 (35.4-47.2)	30.0 (20.7-41.3)	3.5 (1.4-8.9)	25.3 (15.6-38.1)
Retired	776	59.3 (38.8-77.0)	7.6 (5.7-10.1)	4.4 (2.7-7.0)	28.8 (13.2-51.6)
Unable to work	251	38.6 (29.1-48.9)	21.5 (15.5-29.0)	7.7 (5.0-11.8)	32.2 (23.5-42.3)

Abbreviation: CI, confidence interval.

a Estimates are age-standardized to the US 2000 census data.

b Numbers may not equal total because of missing data.

c Determined by responses to questions regarding substance abuse, mental illness, whether mother or stepmother was treated violently, and incarceration of a household member.

d Determined by responses to questions regarding psychological abuse, physical abuse, and sexual abuse.

**Table 3:** Adjusted Odds Ratios^a^ for the Relationship Between Adverse Childhood Experiences (ACEs) and Current Smoking, Obesity, and Fair or Poor General Health, 2002 Texas Behavioral Risk Factor Surveillance System

**ACEs**	Current Smoking^b^	Obesity^c^	Fair or Poor General Health^d^
None	1.0 [Reference)	1.0 [Reference]	1.0 [Reference]
Any childhood abuse^e^	1.2 (0.9-1.6)	1.5 (1.1-1.9)	1.7 (1.3-2.3)
Any household dysfunction^f^	1.4 (1.2-1.8)	1.1 (0.9-1.3)	1.5 (1.1-1.9)
Both	1.9 (1.6-2.4)	1.3 (1.1-1.6)	2.0 (1.5-2.5)

a Adjusted for age, sex, race/ethnicity, education level, and employment status.

b Current smoking was defined as smoking at least 100 cigarettes and now smoking some days or every day.

c Obesity was defined as having a body mass index of ≥30 kg/m^2^.

d Self-reported general health was reported as "fair" or "poor" compared with "excellent," "very good," or "good."

e Determined by responses to questions regarding psychological abuse, physical abuse, and sexual abuse.

f Determined by responses to questions regarding substance abuse, mental illness, whether mother or stepmother was treated violently, and incarceration of a household member.
